# The Preparation of Hydroxyl-Terminated Deproteinized Natural Rubber Latex by Photochemical Reaction Utilizing Nanometric TiO_2_ Depositing on Quartz Substrate as a Photocatalyst

**DOI:** 10.3390/polym14142877

**Published:** 2022-07-15

**Authors:** Apisara Sillapasuwan, Phattharawadi Saekhow, Porntip Rojruthai, Jitladda Sakdapipanich

**Affiliations:** 1 Department of Chemistry and Center of Excellence for Innovation in Chemistry, Faculty of Science, Mahidol University, Salaya Campus, Phutthamonthon, Nakhon Pathom 73170, Thailand; apisara.sia@student.mahidol.ac.th (A.S.); ptrwd.sae@gmail.com (P.S.); 2 Division of Chemical Industrial Process and Environment, Faculty of Science, Energy and Environment, King Mongkut’s University of Technology North Bangkok, Rayong 21120, Thailand; porntip.r@sciee.kmutnb.ac.th

**Keywords:** natural rubber, telechelic low molecular-weight natural rubber, photochemical degradation, titanium oxide (TiO_2_), quartz

## Abstract

Hydroxyl-terminated natural rubber (HTNR) is a product of interest for making natural rubber (NR) easy and versatile for use in a wide range of applications. Photochemical degradation using a TiO_2_ film that has been deposited on a glass substrate is one of the fascinating methods of producing HTNR. Nevertheless, light energy is wasted during the photodegradation process because a glass substrate has a cutoff for ultraviolet light. To enhance the effectiveness of the process, a quartz substrate was coated with the TiO_2_ film for photochemical breakdown. X-ray diffraction (XRD) spectroscopy and atomic force microscopy (AFM) were applied to investigate the TiO_2_ deposited on glass and quartz substrates. In addition, the influence of several factors, such as rubber and surfactant concentrations, on the reaction was investigated. After the reaction, the properties of the rubber products, including intrinsic viscosity, molecular weight, and microstructure, were determined. A unique diffraction peak for the anatase (101) phase could be observed in the TiO_2_ film deposited on the quartz substrate, resulting in photochemical activity and photocatalytic efficiency significantly higher than those of the substrate made of glass. In the scenario of deproteinized NR (DPNR) latex containing 10% DRC, 20% *w*/*w* H_2_O_2_, and TiO_2_ film coated on a quartz substrate, the HTNR could be manufactured effectively.

## 1. Introduction

Natural rubber (NR) is a high molecular-weight (MW) natural material having excellent mechanical properties, such as resilience, tensile and tear strength, and fatigue resistance [1] that make it suitable for use in engineering applications. Moreover, there have been many attempts modify NR latex by reducing its MW and introducing reactive functional groups into the rubber chain-ends using an oxidation reaction to give it specific properties. The low-MW NR also known as liquid NR with functional end groups, called telechelic low-MW NR (TLNR), has been applied for use as a compatibilizer [2,3], adhesive [4], as well as for chain extension [5] and grafting [6]. TLNR with various functional end groups has been prepared, for example, using carbonyl-terminated natural rubber (CTNR) and hydroxyl-terminated natural rubber (HTNR) [7,8,9,10,11].

TLNR has been reported to be prepared by different methods such as mechanical, thermal, chemical, and photodegradation processes [7,8,11,12,13]. Photodegradation is non-toxic, non-acidic, and non-thermal, which makes it environmentally friendly. Titanium dioxide (TiO_2_) is the most widely used photocatalyst in a variety of applications, including antibiotic drugs [14,15], wastewater treatment [16], and UV-sensitive hydrophilic surfaces [17], because of its advantages of low cost, high stability, non-toxicity [18], and high photo-reactivity [19]. Valence band electrons of TiO_2_ are excited by a light source. The light energy makes electrons jump up from the valence band to the conduction band with energy equal to or greater than the band-gap energy of TiO_2_, the conduction-band electrons, and valence-band holes generated on the surface of TiO_2_ particle [20]. In the case of anatase, the band-gap energy is 3.2 eV; therefore, the wavelength of the light source must be less than 388 nm. In addition to being a clean method with low energy consumption, the preparation of TLNR via photochemical degradation of NR in the latex phase is also simplified, economical, and convenient for upscaling. TiO_2_ film coated on glass petri dish is an effective film for the photochemical degradation reaction of NR latex [21]. Our research investigated the preparation of functionalized low-MW NR latex using TiO_2_ from deproteinized NR (DPNR), skim latex, and styrene–butadiene rubber [22]. HTNR prepared from DPNR latex gave a higher content of hydroxyl groups than fresh NR latex and high ammonia NR latex due to the fact that the protein in NR, which acts as a natural stabilizer, blocks the addition of hydroxyl radicals on the particles of NR under reaction [23] Nevertheless, a limitation of the reaction site to generate reactive oxygen species was observed [24]. 

In addition, the glass substrate has a cutoff wavelength in the UV region, resulting in lower energy for photochemical degradation. Ordinary glass partially blocks UV-A (315–400 nm) while almost fully blocking UV-B (280–315 nm) and UV-C (100–280 nm). The fused quartz, a type of glass containing primarily silica in non-crystalline form, does not absorb UV light. Consequently, TiO_2_ film coated on quartz irradiated under a UV lamp can efficiently absorb UV light with a wavelength shorter than 300 nm [25]. The zinc–titanate nanocomposite film coating on quartz substrate exhibited high photocatalytic activity, which was evaluated from the photodegradation of direct blue 71 (DB71) under UV–visible light irradiation [26].

In this research, we aimed to develop a method to prepare HTNR through the photochemical reaction of DPNR latex employing TiO_2_ as a photocatalyst. Methylene blue (MB) was also used to examine how glass and quartz substrates affected the photosensitivity of TiO_2_ film. Characterization was done on the structural and molecular weight changes that occurred in DPNR latexes after photochemical reaction under a variety of conditions, such as concentrations of rubber and sodium dodecyl sulfate (SDS) surfactant.

## 2. Materials and Methods

### 2.1. Materials

Fresh NR latex was kindly provided by Thai Rubber Latex Group Public Co., Ltd. (Samut Prakan, Thailand). Titanium solution (3.5% *w*/*w*) supplied by Mitsubishi Gas Chemical Co., Inc. (Mitsubishi Gas Kagaku Co., Tokyo, Japan) was used as received. Other chemical reagents were analytical grade, i.e., methylene blue (MB), which was supplied by Scharlau; hydrogen peroxide (H_2_O_2_), calcium chloride (CaCl_2_), potassium dichromate (K_2_Cr_2_O_7_), sodium hydroxide (NaOH), and sodium dodecyl sulfate (SDS), which were supplied by BDH; and chloroform (CHCl_3_), formic acid, sulfuric acid (H_2_SO_4_), and tetrahydrofuran (THF), which were supplied by Labscan. Acetone, methanol, and toluene (commercial grade) were supplied by Labscan.

### 2.2. Methods

#### 2.2.1. Preparation of TiO_2_-Depositing Substrate

Both substrates, including quartz and glass slide (2.5 × 7.5 cm), were preliminarily cleaned with methanol, distilled water, and cleansing agent (chromic acid cleansing mixture; a mixture of K_2_Cr_2_O_7_ and conc. H_2_SO_4_) and subsequently rinsed with distilled water. Before drying, they were soaked in 0.1 M NaOH for 30 min and washed again with distilled water. 

To prepare TiO_2_ film, 50 µL of 2.75% *w*/*v* titanium solution was spin-coated on the cleaned substrates at 2000 rpm for 120 s. After being at room temperature for 30 min, the coated substrates were dried at 100 °C for 30 min and finally annealed at 550 °C for 60 min in a furnace at a rate of 2 °C/min to remove residual organic compounds. 

#### 2.2.2. Preparation of DPNR

Fresh NR latex preserved with 0.3% *v*/*v* NH_4_OH solution was incubated with 0.04% *w*/*v* proteolytic enzyme (KP 3939, Kao Co., Tokyo, Japan) and 1% *w*/*v* SDS at 37 °C for 24 h, followed by centrifuging of the mixture twice at 13,000 rpm at 25 °C for 30 min. The cream fraction was finally re-dispersed with 1% *w*/*v* SDS and distilled water to obtain DPNR latex.

#### 2.2.3. Photosensitivity of TiO_2_ Film by MB

A mixture solution of 100 ppm of MB (5 mL), 30% *w*/*v* H_2_O_2_ (5 mL), and distilled water, which was used to adjust the volume to 100 mL, was poured on the TiO_2_ film-coated and non-coated containers. The samples were exposed in a UV chamber containing UV-C lamps (20 W) with a distance of 10 cm between the sample and lamps. The MB mixture was sampled from the same flask at various times and then subjected to UV–Vis absorption measurement at 663 nm, and the concentration of UV-exposed MB was calculated by the calibration curve.

The photosensitivity of TiO_2_ film was tested on the degradation and decolorization process of MB. The concentration of MB was determined by a UV–Vis spectrophotometer at 366 nm. In the presence of both TiO_2_ and H_2_O_2_, MB can absorb UV light energy and convert the passage form, blue pigment, to the sulfoxide form (excited form), which is colorless. The photocatalytic decolorization of MB is a first-order reaction, and its kinetics may be expressed as follows [23]:

ln (C/C_0_) = −*kt* (1)

where *k* is the apparent reaction rate constant, and C_0_ and C are MB’s initial and reaction concentrations, respectively. Note that the calibration curve of MB concentration relating to absorbance was established based on Beer–Lambert’s law. The relationship between absorbance and concentration of methylene blue can be determined from the following equation:

Abs_663_ = 0.1436 C_MB_ + 0.006 (2)

Abs_663_ is the absorbance of MB solution at 663 nm, and C_MB_ is the concentration of MB in ppm. Thus, the percentage of the remaining MB (%MB) as a function of UV irradiation time was investigated.

(3) $$\%\mathrm{MB}=\frac{{\mathrm{MB}}_{t}}{{\mathrm{MB}}_{0}}\times 100$$

where ${\mathrm{MB}}_{t}$ is MB concentration at irradiation time t, and ${\mathrm{MB}}_{0}$ is initial concentration.

#### 2.2.4. Photosensitivity of TiO_2_ Film by DPNR

A total of 5.0 g of 10% DRC of DPNR latex and 20% *w*/*w* of H_2_O_2_ were poured on TiO_2_-coated containers and irradiated under a UV-C lamp in the UV chamber. Then, the reaction was sampled every 1 h until 6 h, followed by subjection to a UV–Vis spectrophotometer. The latex was coagulated with acetone and purified by reprecipitation in methanol. Finally, the sample was dried under vacuum at 40 °C until a constant weight before further MW and intrinsic viscosity determinations.

### 2.3. Characterizations

#### 2.3.1. Crystallinity of TiO_2_ Film Deposited on the Substrates

The crystallinity of the coated TiO_2_ was characterized by X-ray diffraction using a Bruker diffractometer using CuK_α_ radiation (λ = 0.154056 nm) over the range 20 < 2θ < 60°, with a secondary graphite monochromator. The surface topology of TiO_2_ film was determined by atomic force microscopy (AFM) (Nanoscope^®^III, Digital instrument), operating in the contact mode with a scan size of 1.00 µm for a 1 × 1 cm glass slide.

#### 2.3.2. Molecular Weight (MW) and Chemical Structure of the Rubber Samples

The intrinsic viscosity was measured using an Ubbelohde solution viscometer (SCHOTT^®^, CT52). Viscosity-average MW ($\bar{M_{v}}$) was calculated by Mark–Houwink’s equation. The Mark–Houwink constant, *K*, used in this calculation was 33.1 × 10^−5^, and the value of 0.71 was used for the constant ‘*a*’.

Fourier-transform infrared (FT-IR) spectrophotometry was carried out using a JASCO^®^ FTIR-460 plus. The degraded DPNR was cast on a KBr disk and subjected to measurement at a resolution of 4 cm^−1^ with 100 scans to analyze the functional group of samples.

Gel permeation chromatography (GPC) was used to determine the weight-average MW ($\bar{M_{w}}$) and number-average MW ($\bar{M_{n}}$) using a JASCO^®^-Borwin GPC. The dried latex was dissolved in THF to make a solution with a concentration of 0.05% *w*/*v* and filtered through a 0.45 µm nylon membrane to remove impurities and gel in a rubber solution. 

^1^H and ^13^C-NMR spectroscopies were recorded on a BRUKER DPX 300. The rubber of 10–30 mg was dissolved in *d*-chloroform (CDCl_3_) in the presence of 1% TMS as an internal standard.

The morphology of the latex sample was characterized by scanning electron microscopy (SEM) using a HITACHI SEM S-2500 system. The samples were secured onto an aluminum stub and coated with platinum/palladium in a Plaron high.

AFM was used to examine the surface topologies of rubber particles. Topographic information was acquired using a Digital Instruments Nanoscope^®^ IIIa, scanning probe microscope controller version 4.31ce. The sample was prepared by depositing latex onto freshly cleaved glass and dried at room temperature. The tapping mode was employed in the AFM measurement to determine topology.

## 3. Results and Discussion

### 3.1. Characterization and Photosensitivity Determination of TiO_2_ Film

#### 3.1.1. Structure Properties of TiO_2_ Film

**Figure 1:**
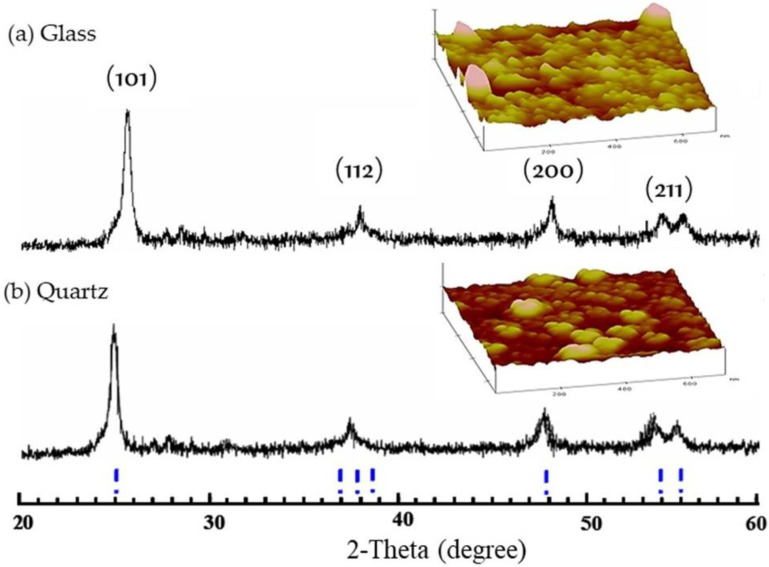
XRD patterns and AFM contact mode topographic images of TiO_2_ film mounted on (**a**) glass, and (**b**) quartz substrates.

The XRD patterns of TiO_2_ films mounted on quartz and glass substrates are presented in Figure 1. The crystallinity of TiO_2_ films coated on both substrates was found to be mainly anatase after annealing, which could be observed from the diffraction peaks (2θ) at 25.3, 38.5, 48.0, and 55.0 degrees, corresponding to (101), (112), (200), and (211), respectively. It has been confirmed that the anatase form has the highest photocatalytic activity among other crystalline forms [27,28]. However, unsuitable film preparation can yield TiO_2_ powder instead of TiO_2_ film, which may affect the TiO_2_ crystallite behavior that depends on the crystallite size grown after annealing at higher temperatures [29]. 

#### 3.1.2. Surface Topology of TiO_2_ Film

The photocatalytic activity of TiO_2_ film is strongly dependent on the crystal structure, crystallite size, thickness, and roughness. From the surface topology of TiO_2_ films coated on two substrate types at a scan range of 1.0 × 1.0 µm, as presented in Figure 1, the mean roughnesses (R_a_) of TiO_2_ films investigated from different areas and different samples were about 2.92 ± 0.26 nm and 2.73 ± 0.36 nm for quartz and glass substrates, respectively, with a mean diameter of about 10 nm for glass and 13 nm for quartz. Moreover, these films showed the rough surface texture of particles fused at the interparticle contacts.

#### 3.1.3. The Transmittance of the Substrate

**Figure 2:**
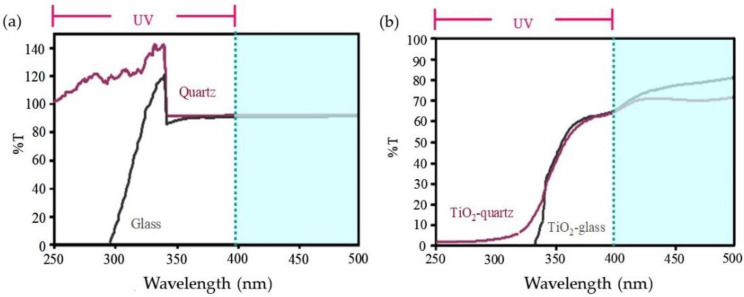
UV–Vis spectra of (**a**) raw quartz and glass, and (**b**) TiO_2_ film deposited on quartz, and glass by repeating 3 coating cycles.

As discussed previously, UV light absorption is a drawback of TiO_2_ film coated on a glass substrate. This study utilized pure SiO_2_ glass, commonly known as fused quartz, which does not absorb UV radiation, as opposed to the more efficient transparent TiO_2_ surface. As depicted in Figure 2a, the optical characteristics of both substrates were evaluated by UV–Vis spectrophotometry in the wavelength range between 250 and 500 nm.

This may be because the fused quartz is made from a silicon-rich chemical precursor usually used in continuous flame hydrolysis. Transparent glass with ultra-high purity is important in producing optical transmission in the deep ultraviolet range [30]. Moreover, a similar result was still observed in TiO_2_ film deposited on quartz, showing higher transparency in the UV range than in the glass, as presented in Figure 2b.

#### 3.1.4. Photosensitivity of TiO_2_ Film on MB 

**Figure 3:**
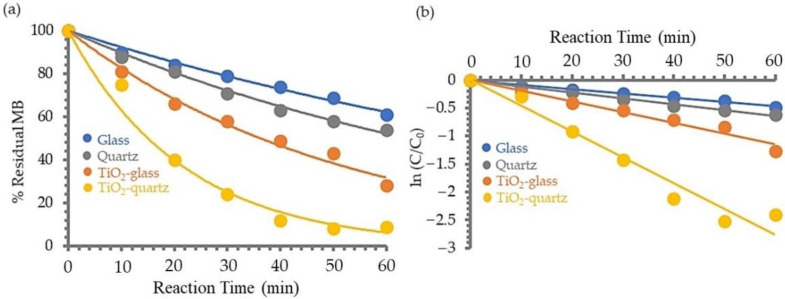
Photosensitivity of TiO_2_ film on MB in the absence and presence of TiO_2_ film coated on glass and quartz substrates: (**a**) photodecomposition of MB, and (**b**) photodecomposition kinetics of MB.

The photodecomposition of aqueous MB in the presence of H_2_O_2_ using TiO_2_ film coated on glass and quartz substrates, prepared by a spin-coating process, was compared to that of MB using the non-coated substrate to determine which substrate was more effective. The results of this comparison are plotted in Figure 3a. It is clear that the amount of MB decreased as the UV irradiation time increased in all cases. Due to the enhanced UV transparency of the quartz substrate, the decomposition of MB was shown to be superior to that of the glass substrate in all cases. Furthermore, the decomposition of MB was greater in the condition using TiO_2_ film coated on both substrates than it was in the absence of one, and the decomposition of MB in the presence of TiO_2_ film coated on quartz was faster than that coated on glass substrates. This result suggests that UV light can pass through quartz to activate TiO_2_ film to react more reactivity in decomposing MB than glass. The relationship between ln (C/C_0_) and *t* (substrate irradiation time) is shown in Figure 3b. The plot gives a fairly good straight line, indicating that the photocatalytic degradation of MB is a first-order kinetic reaction and expressed as Equation (1). The slopes of these lines can be used for calculating the first-order rate constant, *k*. The estimated values of *k* (min^−1^) were 0.0075 (glass), 0.0108 (quartz), 0.0190 (TiO_2_ film on glass), and 0.0460 (TiO_2_ coated on quartz). The photocatalytic activity can be ordered as follows: glass < quartz < TiO_2_ film coated on glass < TiO_2_ film coated on quartz. It can be deduced that using quartz instead of glass substrate could increase the decomposition rate of MB, especially in the case of coating with TiO_2_.

#### 3.1.5. Photosensitivity of TiO_2_ Film on DPNR

**Figure 4:**
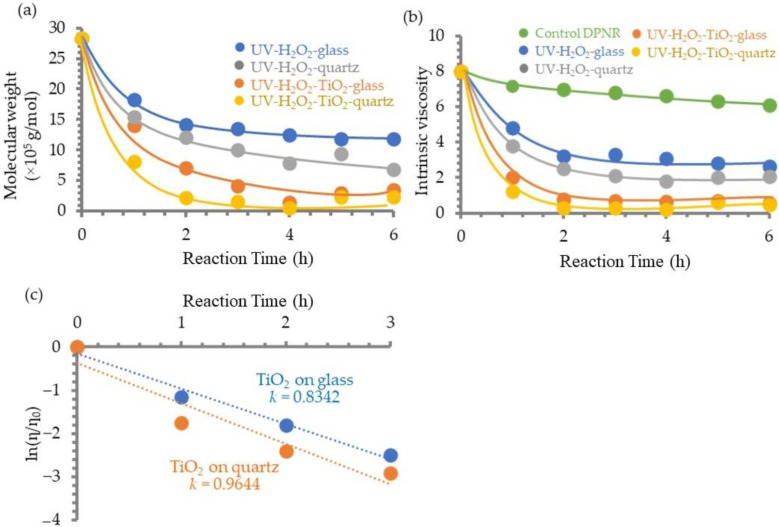
The effect of TiO_2_ film and substrate types on (**a**) the MW and (**b**) the intrinsic viscosity of DPNR and UV irradiation time, and (**c**) the relationship of ln relative viscosity of DPNR and UV irradiation time.

The photochemical reaction of DPNR latex can be carried out by photocatalytic decomposition by H_2_O_2_ under UV irradiation in the presence and absence of TiO_2_ film deposited on glass and quartz. Figure 4a,b show the effect of the photodecomposition on the MW and intrinsic viscosity, respectively, of DPNR latex at various UV irradiation times. Regardless of the circumstances, the MW and the intrinsic viscosity of DPNR latexes decreased as the time spent under UV irradiation increased, particularly in the first two hours. The MW and the intrinsic viscosity of DPNR latexes treated with quartz were lower than those using glass as the substrate. In addition, it was found that they were found to be lower than those treated with glass as the substrate. In the case of the presence of TiO_2_ film coated on the substrates, the intrinsic viscosity of DPNR samples decreased from 8 to 0.8 and 0.3 for glass and quartz, respectively, after two hours of reaction. These results imply that the mounted TiO_2_ film accelerated the efficient photodecomposition of DPNR latex, and the quartz substrate enhanced the reaction competency. 

In order to investigate the efficiency of substrate for the photocatalytic reaction of DPNR latex, the relationship between ln(η_t_/η_0_) and UV irradiation time of both substrates, which were coated with TiO_2_, were plotted as shown in Figure 4c. The relationships were approximately linear for both substrates; consequently, the rate constant, *k*, for DPNR photodecomposition could be calculated as 0.8342 and 0.9644 h^−1^ for glass and quartz, respectively. This evidence indicates that the reaction was conducted more rapidly when the quartz substrate was used. Consequently, TiO_2_ infused on quartz was chosen to act as the photocatalyst for the decomposition of DPNR.

### 3.2. Structural Characterizations of HTNR 

**Figure 5:**
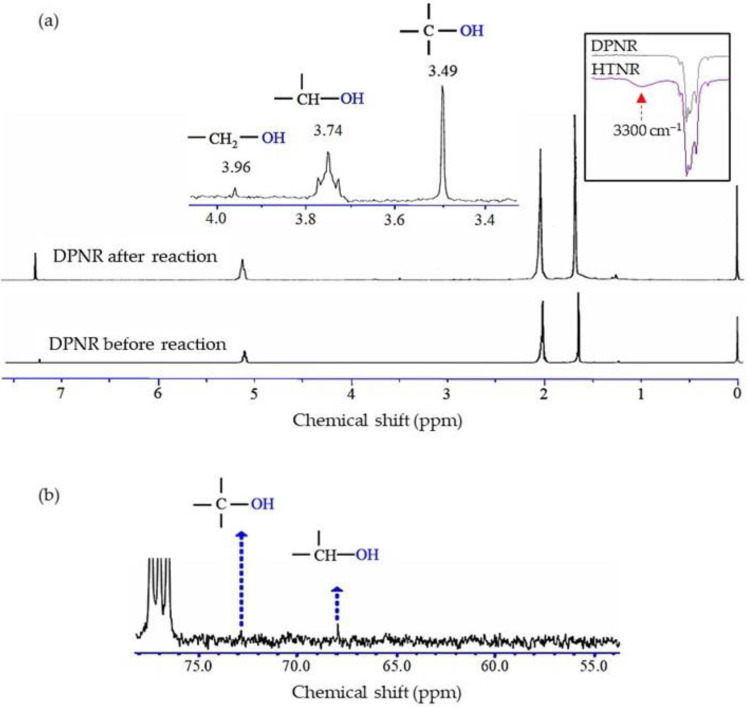
(**a**) ^1^H-NMR and FT-IR spectra of DPNR before and after the photochemical reaction (DPNR and HTNR) under UV for 6 h with TiO_2_ film deposited on quartz substrate, and H_2_O_2_ (**b**) ^13^C-NMR spectrum of DPNR after the photochemical reaction.

The photodegradation of *cis*-1,4-polyisoprene occurred through chain scission under UV irradiation. The three radical species reacted with hydroxyl radicals in the three possible pathways [19], which were generated from the TiO_2_ film surface and the decomposition of H_2_O_2_ to produce the HTNR. The structure of DPNR latex after being subjected to the photochemical reaction by using TiO_2_ film and H_2_O_2_ was confirmed by FT-IR and NMR measurements. Figure 5a represents the ^1^H-NMR and FT-IR spectra of DPNR before and after the photochemical reaction in the presence of TiO_2_ film coated on quartz substrate and H_2_O_2_ after UV irradiation 6 h. From the FT-IR spectra, DPNR after the reaction or HTNR showed a transmittance band around 3300 cm^−1^ corresponding to the hydroxyl group, which could not be detected in the case of the DPNR before the reaction. The presence of hydroxyl groups was due to the addition of hydroxyl radicals under UV irradiation, which came from the decomposition of H_2_O_2_ and the formation of hydroxyl radicals on the surface of TiO_2_ film.

The ^1^H-NMR spectra of DPNR showed that other than the main signal due to the main chain of polyisoprene, the small signals at the chemical shifts of 1.25, 3.49, 3.74, and 3.96 ppm were observed in the rubber products after the photochemical reaction. These small signals could be assigned to methylene protons, protons of hydroxyl group, hydroxylated methine protons, and hydroxylated methylene protons, respectively. The result of the ^1^H-NMR spectrum was consistent with that from the ^13^C-NMR spectrum of DPNR after the reaction, exhibiting small signals at the chemical shifts of 67.98 and 72.84 ppm, corresponding to tertiary and methine carbon attached to the hydroxyl groups, respectively, as shown in Figure 5b. These signals were not observed in the case of the DPNR before subjection to UV irradiation. According to the finding presented above, the low-MW NR with a hydroxyl group at the chain-ends, known as the HTNR, was persuaded to be the product of the photochemical reaction of DPNR in the presence of TiO_2_ film coated on quartz substrate and H_2_O_2_ through UV irradiation exposure for a period of 6 h.

### 3.3. Effect of Latex Concentration (%DRC) 

**Figure 6:**
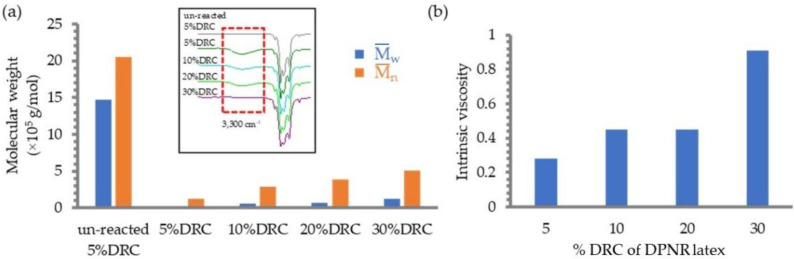
Effect of the latex concentrations on (**a**) the MW and the change of FT-IR spectra, and (**b**) intrinsic viscosity of DPNR after the reaction in the presence of H_2_O_2_ and TiO_2_ film irradiated with UV light for 5 h.

The photochemical reaction of DPNR latexes containing various concentrations with TiO_2_ film deposited on a quartz substrate in the presence of H_2_O_2_ followed by UV irradiation for 5 h at pH 5 was investigated. The FT-IR spectra of DPNR after the reaction showed a broad band around 3300 cm^−1^ corresponding to the –OH group, as presented in Figure 6a. The amount of –OH groups generated on rubber was calculated by the ratio of band areas of –OH groups per methyl group (CH_3_) at 1450 cm^−1^ in TLNR at various latex concentrations after the reaction. The band area ratios of –OH/–CH_3_ were found to be 0, 0.015, 0.1308, 0.1452, and 0.2297 for un-reacted DPNR latex (control), 30, 20, 10, and 5% DRC, respectively, showing increases of the ratio with decreases of %DRC. This suggests that the increase in latex concentration resulted in a decreased number of –OH groups on the rubber chain. In other words, the photochemical reaction was successfully carried out and contributed to a greater number of –OH groups on the rubber chain at the lower DPNR latex concentration. However, if the latex concentration is too low, the cost will be much higher. DPNR latex should have a concentration of 10% according to this formula.

Figure 6a,b show the change in MW and intrinsic viscosity at various latex concentrations between 5 and 30% DRC. It was discovered that an increase in the latex concentration led to an increase in both the MW and the intrinsic viscosity of the latex. This result indicates that the reaction took place under conditions with lower latex concentrations, which resulted in the production of DPNR samples with a terminal hydroxyl group having a low MW. This finding conforms to the FT-IR result that showed an increase in the band area of the hydroxyl group.

### 3.4. Effect of Surfactant 

The effect of surfactant on the photochemical reaction of DPNR latex was investigated by varying the concentration of SDS in the experiment. SDS is an anionic surfactant that is typically utilized in order to stabilize the DPNR particles. The hydrocarbon tails of SDS embed themselves in the rubber particles, making the hydrophilic head of SDS accessible to water. 

**Figure 7:**
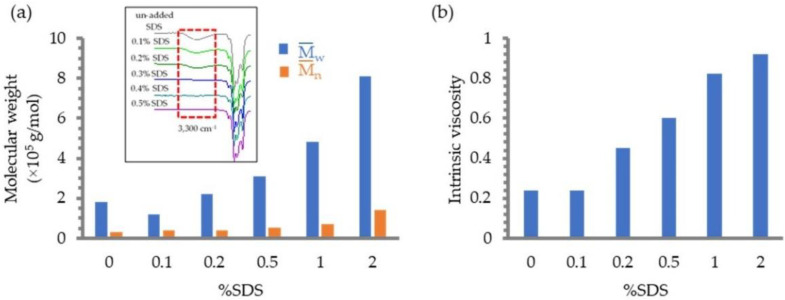
Effect of the surfactant concentrations on (**a**) the MW and the change of FT-IR spectra and (**b**) intrinsic viscosity of DPNR after the reaction in the presence of H_2_O_2_ and TiO_2_ film irradiated with UV light for 5 h.

The MW and intrinsic viscosity of DPNR latexes after subjection to photochemical degradation with various SDS concentrations are shown in Figure 7a,b, respectively. At a low concentration of SDS, approximately 0.1%, the MW and the intrinsic viscosity of the degraded DPNR were comparable to those of the DPNR latex without the addition of SDS and rose dramatically as the concentration of SDS increased. Based on the FTIR data, the findings support previous findings that the intensities of hydroxyl groups have decreased (Figure 7a). It is common knowledge that the molecules of SDS applied to latex can encircle the surface of the rubber to serve as a stabilizing force for the particles. Consequently, it is conceivable to hypothesize that when subjected to a high concentration of SDS, the rubber particles should be enveloped by an arrangement of SDS molecules that is as dense as possible. This may result in the rubber particles being protected from the addition of hydroxyl radicals formed in the aqueous phase of latex. In addition, the extra SDS could form micelles of varying sizes, which would result in a decrease in the intensity of the UV radiation necessary to break the C–C bond. In addition, the hydroxyl radical might be contained within these micelles, reducing the total quantity of radical that needs to be added to the rubber chain.

### 3.5. Study the Morphology of DPNR Particles by Scanning Electron Microscopy (SEM) 

**Figure 8:**
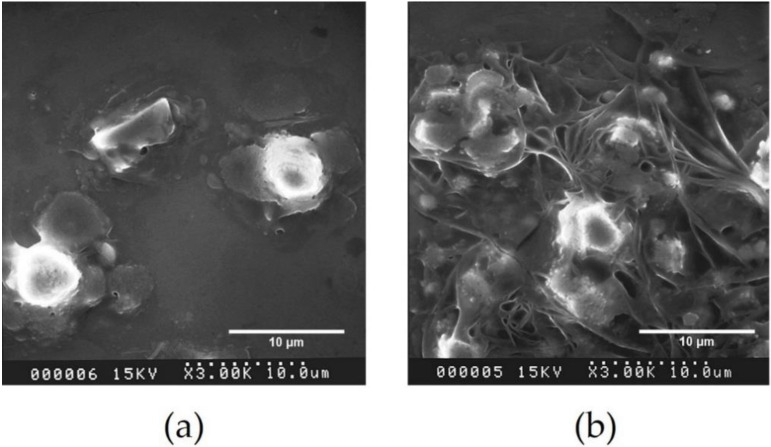
Scanning electron micrographs of DPNR particles (**a**) before and (**b**) after the photochemical process with 20% H_2_O_2_ and TiO_2_ film irradiated under UV light for 5 h on the quartz substrate.

Figure 8a is a scanning electron micrograph (SEM) of rubber particles taken before the photochemical process began. In this micrograph, the rubber particles’ topology cannot be noticed. The rubber particles had a spherical shape, and their diameters were approximately 4 μm. It was clear that the particle size of DPNR latex was significantly larger than the standard size of rubber particles (around 1 μm). It is likely that the latex was not sufficiently stabilized, which led to the partial coalescence and aggregation of rubber particles. In addition, the shape distortion of particles was noticed after they had been subjected to the photochemical process, as shown in Figure 8b. This was caused by the collapse of the surfaces of the rubber particles.

### 3.6. Characterization of the Morphology of the DPNR Particles by AFM 

**Figure 9:**
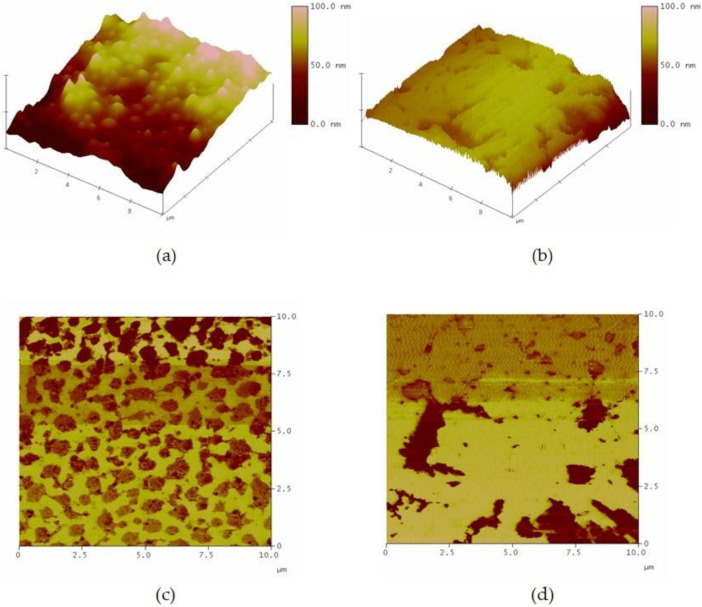
AFM topology (height image and phase image) of DPNR particles (**a**,**c**) before the photochemical process, and (**b**,**d**) after the photochemical process with 20% H_2_O_2_ and TiO_2_ film coated on quartz substrate under UV light for 5 h.

AFM in tapping mode characterized the rubber particles before and after processing via a photochemical reaction. Figure 9a,c and Figure 9b,d represent the AFM topologies and phase-contrast images of DPNR particles before and after the photochemical reaction, respectively. The AFM image demonstrates that the rubber particles kept their spherical shape before subjection to the photochemical reaction; however, the rubber particles appeared united after the photochemical process, forming a continuous phase as a relatively homogeneous matrix. The modifications in surface topology demonstrated that the photochemical reaction facilitated the coalescence of rubber particles, hence promoting film formation. In addition to this phenomenon, the roughness of the film was decreased from 38.092 to 12.216 nm during the process. This evidence may be the result of polar interactions between hydroxyl groups on the rubber chain and the quartz substrate, which led to the flattening of the surface of the film.

## 4. Conclusions

The HTNR was achieved through the photochemical reaction of DPNR latex with a TiO_2_ film that was deposited on a substrate in the presence of H_2_O_2_. The anatase TiO_2_ coated on quartz utilized as a photocatalyst gave a higher efficiency in the breakdown of DPNR latex compared to that on glass substrate. This evidence was demonstrated by the faster reaction and the lower MW of rubber products. FT-IR, ^1^H, and ^13^C-NMR spectroscopies were all able to support the structure of HTNR. In part, the low photochemical reaction rates could be attributed to the high concentrations of both latex and surfactant. As a result, the HTNR was successfully synthesized utilizing a TiO_2_ film mounted on a quartz substrate in a photochemical reaction with 10% DRC of DPNR latex and 20% *w*/*w* H_2_O_2_.

## Data Availability

Not applicable.
